# Optimization of the dosage regimen of zoledronic acid with a kinetic-pharmacodynamic model and exposure-response analysis

**DOI:** 10.3389/fphar.2023.1089774

**Published:** 2023-09-27

**Authors:** Huan Wang, Qi Liu, Muhan Jiang, Chunli Song, Dongyang Liu

**Affiliations:** ^1^ Department of Orthopedics, Peking University Third Hospital, Beijing, China; ^2^ Drug Clinical Trial Center, Peking University Third Hospital, Beijing, China; ^3^ Center of Clinical Medical Research, Institute of Medical Innovation and Research, Peking University Third Hospital Beijing, Beijing, China

**Keywords:** zoledronic acid, osteoporosis, dosing regimen, kinetic-pharmacodynamic model, exposure-response analysis

## Abstract

**Purpose:** In order to support the dose optimization of zoledronic acid, the kinetic-pharmacodynamic model and exposure-response analysis were used to describe the changes in bone mineral density in different doses of zoledronic acid and establish the relationship between dose and acute phase reaction.

**Methods:** Data were extracted from literature in accessible public databases. The kinetic-pharmacodynamic model was developed based on the above data using the NONMEM package to estimate parameters describing the relationship between the dose of zoledronic acid and bone mineral density. Exposure-response analysis was developed to establish the relationship between dose and acute phase reaction. Model evaluation was performed using goodness-of-fit, coefficient of variation (CV%). And sensitivity analyses were performed to assess the necessity of related parameters. Then the established model was used to simulate the changes of bone mineral density under different administration regimens, and the literature data was verified.

**Results:** The kinetic-pharmacodynamic model successfully described zoledronic acid dose and change of bone mineral density in osteoporosis patients, with coefficient of variation of most less than 71.5%. The exposure-response analysis showed the incidence of acute phase reaction is dose-dependent. The bone mineral density was simulated based on the developed kinetic-pharmacodynamic model. And the simulated change of bone mineral density and the incidence of acute phase reaction could be helpful to propose a dosage regimen.

**Conclusion:** Overall, the kinetic-pharmacodynamic model described changes of bone mineral density in different doses of zoledronic acid *in vivo*. And, the model and the exposure-response analysis also showed to provide the assessment of dose-response relationship for zoledronic acid.

## 1 Introduction

Osteoporosis (OP) is a chronic disease characterized by low bone mass and microarchitectural deterioration of bone tissue, resulting in increased bone fragility and susceptibility to fracture and even disability or reduced life (Muñoz et al., 2020). Its prevalence in the world was reported to be 18.3% (Salari et al., 2021). It has become the fourth most common chronic disease after hypertension, diabetes, and coronary heart disease. This is especially true for the elderly population [more than 10 million US patients >50 years old and half of the Chinese population >75 years old had OP (Guzon-Illescas et al., 2019)]. As the world enters an aging society, it is expected that by 2050, the incidence of osteoporosis will be two to three times higher than it is today (Wang et al., 2021).

Bisbisphosphonates (BPs) are currently the first-line therapy for the treatment of osteoporosis (Johnston and Dagar, 2020). Among them, zoledronic acid is the third generation of BPs used to treat primary osteoporosis, secondary osteoporosis or low bone mass worldwide. Compared with the first- and second-generation BPs, zoledronic acid has a stronger binding ability with bone mineral and inhibits bone resorption (Dunford et al., 2001). The anti-resorption effect of zoledronic acid is the strongest among BPs. Annual intravenous injection of zoledronic acid 5 mg can reduce the risk of fracture with osteoporosis by 35%–70% and reduce the mortality rate after hip fracture by 28% (Lyles et al., 2007).

However, the scientific dose of zoledronic acid for osteoporosis is controversial. First, the earliest use of zoledronic acid for the treatment of osteoporosis was to follow the usage and dosage of Paget’s disease indications in the Caucasian population (Armas and Recker, 2012; Appelman-Dijkstra et al., 2018). Second, even in the Caucasian patient population, the dose-exposure-response (D-E-R) relationship has not been studied, and the optimal dosing regimen for osteoporosis has not been determined. Moreover, zoledronic acid is a drug with high affinity for hydroxyapatite, and its dose is directly related to the size of the backbone. The results of the clinical trial of risedronate in the Japanese population suggested that there may be racial differences in its clinical use among Asian and Caucasian populations (Shiraki et al., 2003). In addition, the multiple dose administration of zoledronic acid has a tolerance phenomenon, and the use of zoledronic acid is often discontinued after 3 years of use due to drug holidays (Shiraki et al., 2003). Therefore, it is speculated that the scientific dose of zoledronic acid in the treatment of osteoporosis cannot withstand scrutiny.

In addition, acute phase reaction (APR) often occurs after intravenous administration of zoledronic acid, which forces patients to switch to other anti-osteoporotic drugs (Saag et al., 2007). APR is the most common adverse reaction when zoledronic acid is used for the first time, mainly including fever, headache, skeletal muscle pain and other flu-like symptoms (Lyles et al., 2007). The incidence of APR after the first administration is approximately 24.5%–70.0% (Reid et al., 2010; Ding et al., 2017; Ferreira et al., 2021). Such a high incidence will cause patients to have certain concerns about their safety, and compliance with drug treatment will be reduced. Approximately 26.2% of patients will not continue to receive zoledronic acid treatment (Saag et al., 2007). However, if medication adherence was <80%, the corresponding fracture rate would increase by 17.0% (Lewiecki, 2008; Silverman and Gold, 2010). Studies showed that the incidence of APR was dose-dependent in Paget’s disease, suggesting that dose reduction would effectively reduce the incidence and degree of APR.

The key to optimizing the dose is to establish the D-E-R relationship of zoledronic acid and then to determine the lowest effective dose and the effective drug concentration in bone tissue. Zoledronic acid has a strong bone-binding ability. Compared with the drug concentration in blood, the drug concentration in bone tissue would more directly reflect the effect of its pharmacological action. However, it is difficult to obtain bone tissue in clinical practice, there is a lack of drug concentration data in bone tissue, and it is impossible to elucidate the changes in drug concentration in bone tissue, so the quantitative relationship between bone tissue drug concentration and drug efficacy cannot be confirmed. Traditional clinical trials optimize the dose of a drug, either from a clinical end point (fracture incidence) or from a surrogate measure (bone mineral density or new fractures). There are problems of large interindividual variability (more than 50%), large sample sizes (at least thousands of cases), and long observation times.

Quantitative pharmacological studies can establish the quantitative relationship between dose and response (D-R), and the D-R data of zoledronic acid are sufficient. Therefore, quantitative pharmacological methods for dose optimization have become the most ideal and efficient methods. Although a dose-bone turnover markers (BTMs) - bone mineral density (BMD) model has been established after a single dose (Pillai et al., 2004; Mori et al., 2018; Wu et al., 2021), the tolerance model has not been integrated, there is an error in predicting multiple doses, and dose optimization has not been performed in combination with APR. In addition, the existing models all use one or two BTMs to drive BMD to evaluate the change in BMD. However, there are many factors that affect BMD, so there may be errors when directly using BTM to drive the change in BMD. Thus, this study aims to simulate the changes in BMD under different administration regimens and establish the relationship between dose and APR to support the dosing regimen of zoledronic acid.

## 2 Methods

### 2.1 Database development

A comprehensive search of clinical trials was conducted using PubMed, EMBASE, and ClinicalTrials.gov website. The search keywords were as follows: zoledronate, zoledronic acid, osteoporosis, clinical trial, bone mineral density, BMD, acute phase response, acute phase reaction and APR. The retrieval time of reports was from 01 January 1990, to 30 June 2022.

The inclusion criteria were as follows: 1) randomized controlled clinical trials of zoledronic acid; 2) trials including patients who were diagnosed with primary osteoporosis; 3) trials simultaneously reporting lumbar spine (LS) and total hip (TH) BMD data measured by dual-energy X-ray absorptiometry (DXA); and 4) trials analyzing the incidence of APR under different dosage regimens. The exclusion criteria were as follows: 1) the literature did not clearly describe the dosage; and 2) BMD was assessed by another method instead of DXA.

The risk of bias in the included articles was assessed by the Cochrane Collaboration’s tool in advance. For each eligible study, relevant data were extracted, including groups, dose, number of patients, time, BMD, and subject characteristics. The data in the references were extracted using Graph Digitizer (GetData, Version 1.9). The dose regimen BMD data and population characteristics for analysis from the literature were summarized in Table 1.

**TABLE 1:** Study characteristics for each clinical trials.

ID	Patients	Regimen	Percentage of female(%)	BMI	Weight(kg)	Age	Baseline of vertebral BMD	
1	180	receive a single administration of one of three doses of zoledronic acid 1 mg, 2.5 mg, or 5 mg	100	/	66.3	65.3	1.03	Grey et al. (2014)
2	180	receive a single administration of one of three doses of zoledronic acid 1 mg, 2.5 mg, or 5 mg	100	/	66.3	65.3	1.03	Grey et al. (2012a)
3	616	receive annual intravenous zoledronic acid 5 mg and followed for 6 years	100	25.3	/	75.5	0.81	Black et al. (2012)
4	3,875	receive annual intravenous zoledronic acid 5 mg and followed for 3 years	100	25.1	/	73.1	0.79	Black et al. (2007)
5	175	receive annual intravenous zoledronic acid 5 mg and followed for 2 years	100	22.7	59.3	57.2	0.64	Liang et al. (2017)
6	89	receive a single administration of one of zoledronic acid 5 mg	100	28.2	/	85.4	0.93	Greenspan et al. (2015)
7	458	receive a single administration of one of zoledronic acid 5 mg	100	23.4	56.8	64.3	0.75	Li et al. (2022)
8	330	receive annual intravenous zoledronic acid 5 mg and followed for 2 years	93.6	23.4	52.4	74.0	0.66	Nakamura et al. (2017)
9	20	receive a single administration of one of zoledronic acid 5 mg	100	/	68.0	62.0	1.06	Grey et al. (2012b)
10	91	receive a single administration of one of three doses of zoledronic acid 1 mg, 2.5 mg, or 5 mg	100	/	65.5	63.3	1.03	Grey et al. (2022)

### 2.2 Model development

**FIGURE 1:**
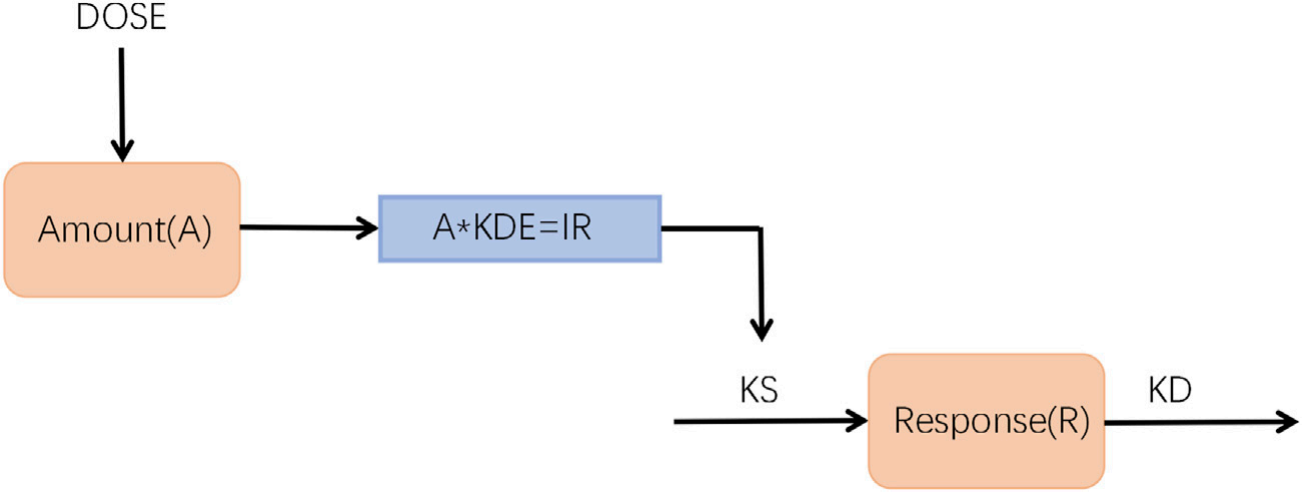
Schematic representation of the K-PD model used to describe the time course of BMD-time profiles after intravenous administration.

The kinetic-pharmacodynamic (KPD) model was developed to describe the BMD-time course of zoledronic acid in different dosages (Figure 1). In the present model, the change in BMD was directly driven by the drug amount, the hypothetical site where zoledronic acid is stored until the onset of the stimulative effect, and the synthesis rate constant KS of the response (R) was inhibited by a virtual infusion rate (IR), expressed in drug amount (A) per time unit, through an Emax model.

$$\frac{dA}{dt}=-KDE\times A$$ (1)

IR=KDE×A (2)

$$\frac{dR}{dt}=KS^{\prime }-KD\times R\;R(0)=\frac{KS}{KD}=\text{BASE}$$ (3)

KS=BASE×KD (4)

$$KS^{\prime }=KS\times \left(1+\frac{IR}{EDK50+IR}\right)$$ (5)

where KDE represents the elimination rate constant from the virtual compartment; KS and KD are the zero-order synthesis and the first-order degradation rate constants of the response R; R(0) is the baseline value of the response before drug administration; IR is the rate of dose-driving; EDK50 represents IR that leads to 50% stimulus of KS; KS' is the new, time dependent, synthesis rate constant observed after drug administration by using the Emax model.

$$\text{Tol}=e^{-k\cdot time}$$ (6)

To account for the observed tolerance of the BMD, an additional variable was included in the model, which was represented in Eq. 6 with the use of first-order time-delay constants (k) on the effect.

Then, Eq. 7 can be expressed as follows:

$$\frac{dR}{dt}=KS^{\prime }\times Tol-KD\times R$$ (7)

### 2.3 Residual analysis

The difference (η) between individual PD parameter estimates and their population estimates were used to describe unexplained random variation in PD parameters, assuming that η follows a (0, ω2) normal distribution. ω2 reflects the unexplained interindividual variability (IIV) or interoccasion variability (IOV) of PD parameters. The difference (ε) between the observed concentration value at each time point and the predicted concentration value calculated from the individual PK parameters describes the random error of the drug concentration, assuming that ε follows a (0,σ2) normal distribution. The interindividual variability model equation and intraindividual variability model equation are as follows:

Kei=TVKe+ηi (8)

where Kei is the individual predicted value of Ke of the i individual, TVKe is the group predicted value of Ke, and represents the difference between the individual predicted value and the population predicted value of Ke, which obeys a (0, ω2) normal distribution, where ω is a constant.

### 2.4 Covariate model

Covariate analysis was used to explore the source of zoledronic acid variation. Model parameters that estimate interindividual variability should consider covariate effects. After establishing the final base model, simple regression can be used to analyze the correlation between each covariate and pharmacokinetic parameters. Age, sex, body mass index (BMI), and body weight were selected as potential covariables of the model structure through a preliminary literature survey. To prevent the influence of collinearity and confounding factors in the covariate model, we used R software to draw box and whisker plots of variables against categorical variables to evaluate the relationship between covariables and parameters. For covariates with high correlation (correlation coefficient >0.8), only one covariable was reserved for model evaluation based on the possibility of a two-parameter correlation mechanism and correlation degree. Finally, the covariates were included in the model, and the SCM function of nonlinear mixed-effect modeling (NONMEM) software was used to automatically screen the covariates by the stepwise method (forward selection: OFV decrease>6.63, α = 0.01, df = 1; backward elimination: OFV increase>10.83, α = 0.001, df = 1).

### 2.5 Model evaluation

After model establishment, the goodness-of-fit (GOF) plots, coefficient of variation (CV%), and precision of the parameter estimates were used to describe the accuracy of the final model. Then, the predictive performance of the final model was evaluated by a visual predictive check (VPC, 2.5th, 50th, and 97.5th percentiles). In addition, bootstrap (N = 500) was used to evaluate the accuracy of parameter estimation. The standard error, shrinkage, and 90% confidence interval (CI) of the parameter estimation of the final bootstrap simulation method were reported and compared with the parameter estimation of the final model.

### 2.6 Exposure-response (E-R) analysis

The clinical trials of zoledronic acid on the incidence of APR were collected, and E-R analysis was used to establish the relationship between the dose and the incidence of APR. According to the different dose, the patients were divided into the 1 mg group, 4 mg group and 5 mg group. Due to the variety in clinical symptoms of APR, APR was not subdivided according to clinical manifestations. The incidence rates of APR in each dose group were calculated and represented by histograms.

### 2.7 Model simulation

**FIGURE 2:**
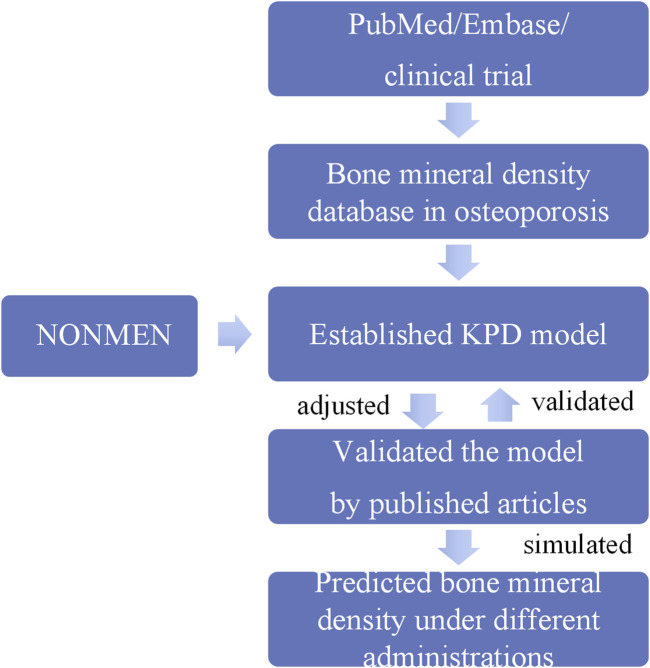
The overall prediction strategy.

Based on the final KPD model, 1,000 simulations were conducted to generate the drug response at the different time points of different dosage regimens, including single-dose and multiple-dose administration. The simulation lasted for 72 months. The results at month 72 were visualized as the median and the 2.5th and 97.5th percentiles. The overall prediction strategy was shown in Figure 2.

### 2.8 Model validation

The final model was used to simulate the drug administration in the literature. Then, the measured values in the literature were used to verify the simulated values.

### 2.9 Software and platform

The majority of literature searches were accomplished by Endnote (Version X7, Thomson Reuters, United States). Data analysis was performed using R programming (Version 3.6.2) and R Studio (Version 1.1.453). Graphical data were extracted via GetData Graph Digitizer. The KPD model was performed using NONMEM (Version 7.2.0, ICON Development Solution, Ellicott City, MD, United States). The first-order conditional estimation with interaction method (FOCE-I) was used for all analyses.

## 3 Results

### 3.1 Characteristics of the included studies

The analysis of this study included a total of 454 trials. All trials were screened according to the inclusion criteria, and 10 studies were eventually included. The detailed dosage and BMD of zoledronic acid were extracted from these 10 published studies (Table 1). These trials in osteoporosis women and men contained 6,014 individuals. The dose of zoledronic acid ranged from 1 mg/year to 5 mg/year with a single dose and multiple doses. Their follow-up period ranged from 12 months to 72 months.

### 3.2 Model development and evaluation

The KPD model established in this study could well describe the dose‒response relationship and the change in BMD after different administrations. The parameters of KPD were presented in Table 2. Zoledronic acid acts on human bones, inhibiting bone resorption by acting on osteoclasts, thereby increasing bone density. Based on the mechanism of action of zoledronic acid, the increase in BMD formation rate (KS) in response to zoledronic acid is modeled with a stimulated Emax model, in which the independent variable is the virtual dose-driving rate (IR). EDK50 represents the IR that leads to a 50% stimulus of KS. During multiple dose administration, the change in BMD was observed to be time dependent. To systematically describe the change in BMD, a time-dependent formula was added to the model, and the observed value and predicted value could be completely fitted. Considering the effect of zoledronic acid on increasing BMD, the time-dependent equation was placed on KS’. This suggested that osteoclast sensitivity to zoledronic acid may be reduced with multiple doses, resulting in reduced receptor synthesis.

**TABLE 2:** Parameter estimates of the KPD model in zoledronic acid.

Parameter(units)	Estimate	RSE (%)	Bootstrap		Shrinkage (%)
			Median	95%CI	
KDE (1/month)	0.08150	71.5	0.12844	0.01812–1.08467	NA
KD (1/month)	0.00474	17.9	0.00516	0.00385–0.00697	NA
EDK50 (ng/month)	41300.0	40.9	28925.7	335.7–108289.8	NA
K	0.00754	20.3	0.00674	0.00001–0.01569	NA
ETA of KDE	FIXED	NA	NA	NA	NA
ETA of KD	FIXED	NA	NA	NA	NA
ETA of EDK50	0.45410	66.4	0.40691	0.00001–2.54713	18.7
ETA of K	FIXED	NA	NA	NA	NA
EPS	0.00005	25.8	0.00003	0.00003–0.00006	7.1

The model was evaluated by GOF plots (Supplementary Figure S1) and the precision of parameters, and sensitivity analysis was performed on parameters with CV (%) less than 71.5. The GOF plot included the diagnostic curve of the group predicted value (Prediction, PRED, Supplementary Figure S2), the individual predicted value (Individual Prediction, IPRED, Supplementary Figure S3) and the actual observed value, as well as the distribution diagnostic curve of the weighted residuals (WRES). The GOF plot showed no large bias, indicating that the model was able to accurately describe the BMD profile of subjects’ zoledronic acid, and plots showing observations versus population and individual predictions showed trends consistent with the unity line. The shrinkage of the parameters was less than 30, and the condition number of the KPD model was 41.83. The VPC results for the KPD model was shown in Supplementary Figure S4. The open circles represent the observations, the solid lines represent the 5th, 50th, and 95th percentiles of the observations, the dashed lines represent the 5th, 50th, and 95th percentiles for the model-based simulated data, and the shaded areas represent the 95% CI for the corresponding quantiles of the simulated data. These VPC plots indicated an adequate predictive ability of the final model. The median of the 500 bootstrap results was close to the final model parameter estimates, and the 95% confidence interval contained the final model parameter estimates, indicating that the final model was robust and the parameters estimate were accurate. The bootstrap results were shown in Table 2. Therefore, these data and plots showed that the model fits the data adequately at the population and individual levels.

During the covariate analysis of zoledronic acid, we searched the literature for reported covariates (age, sex, BMI, etc.). Each was added sequentially (base model), and the developed model was evaluated for its effect on OFV (corresponding to *p* = 0.001) and interindividual variability (IIV) of the parameter estimates. At present, no covariates associated with it were filtered out.

### 3.3 Exposure-response analysis

**FIGURE 3:**
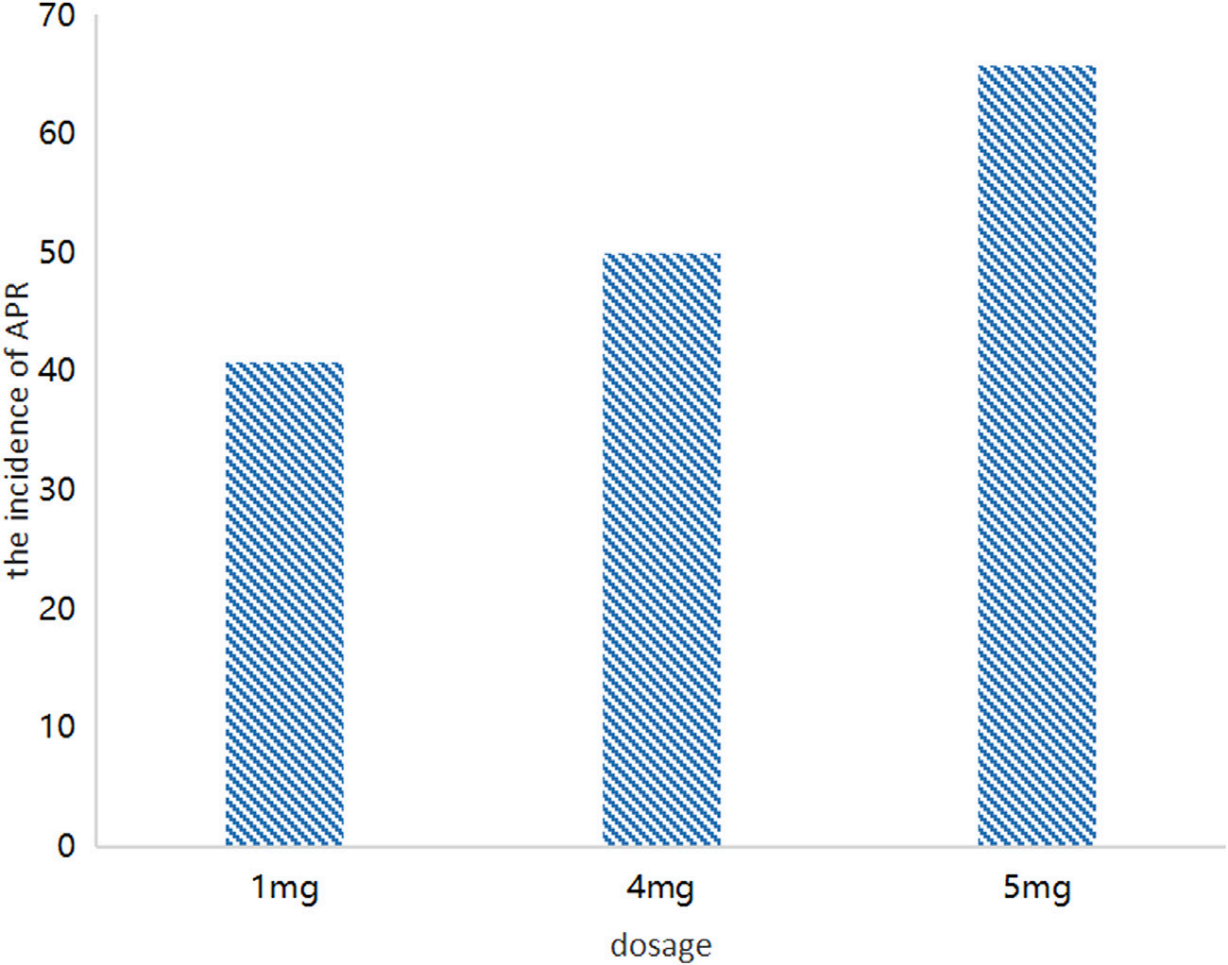
The incidence of APR at different dosages.

We investigated the literature and collected the incidence of APR in patients after administration. The exposure response analysis of APR in different doses supported the optimization of the dose of zoledronic acid, and the results were shown in Figure 3; Table 3. In the dose range of 1 mg–5 mg, the symptoms of APR were dose dependent. The trend of APR also showed that with increasing doses of zoledronic acid, the incidence of APR also increased. The incidence of APR was 40.0%, 50.0% and 65.7% at the dosages of 1 mg, 4 mg and 5 mg, respectively.

**TABLE 3:** Incidence of APR by treatment group.

1 mg			4 mg			5 mg		
APR(N)	Total(N)		APR(N)	Total(N)		APR(N)	Total(N)	
11	27	Grey et al. (2012a)	6	12	Shiraki et al. (2017)	27	20	Grey et al. (2012b)
						109	157	Popp et al. (2017)
						69	127	Sieber et al. (2013)
						146	227	Li et al. (2022)
						158	231	Li et al. (2022)
						6	12	Shiraki et al. (2017)

### 3.4 Model simulation

To support dose optimization of zoledronic acid, we performed simulation studies on the final model. Through model simulation, the changes in BMD under different dosing regimens were obtained.

**FIGURE 4:**
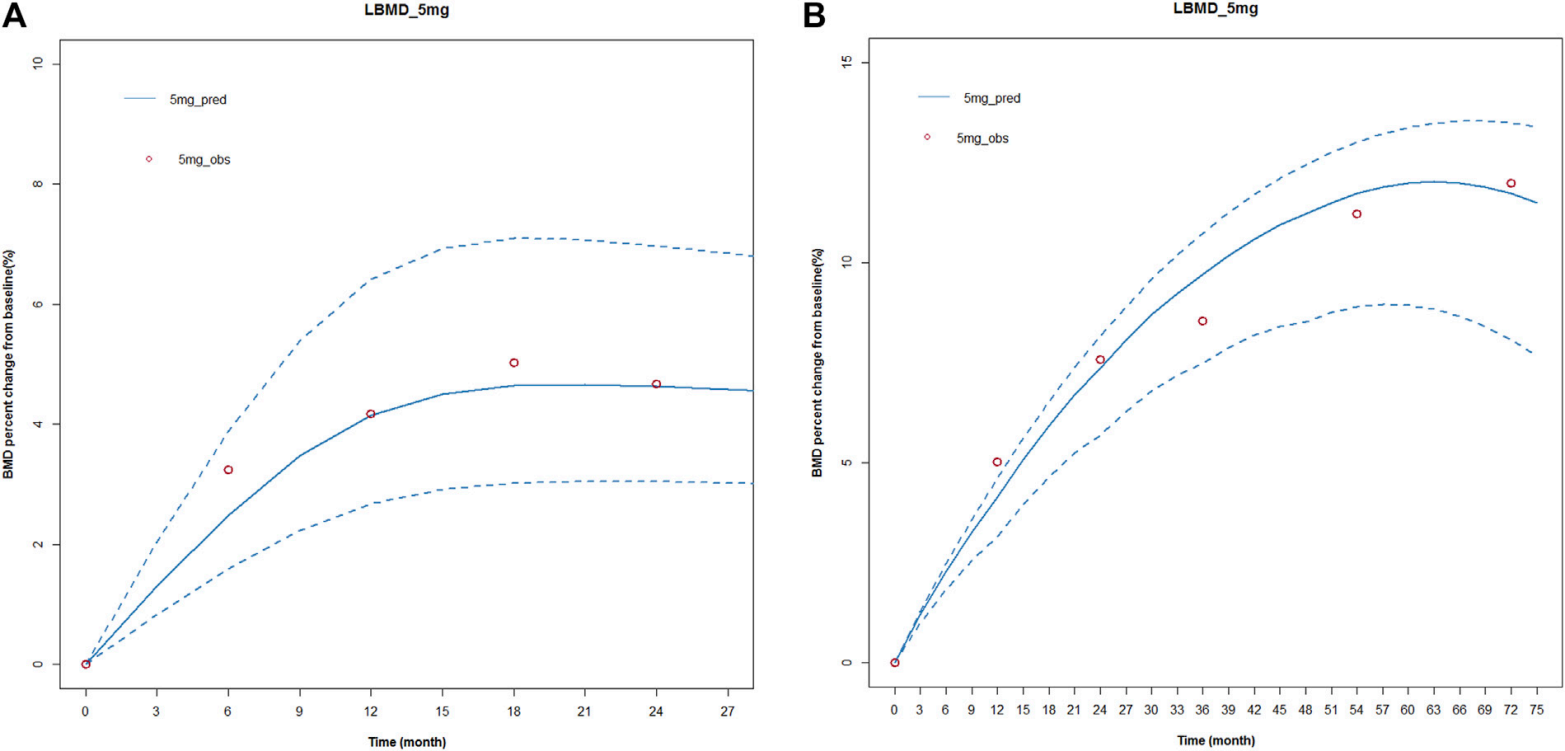
Simulated and fitted BMD profiles of zoledronic acid in patients with osteoporosis after different dosing regimens. **(A)** Single dose; **(B)** multiple doses. **(A)** Single dose: the red circles represent the median observed data from the literature; the blue solid line presents simulated data, and the blue dotted line represents the simulated 95 percent confidence interval. A single-dose regimen was administered once and observed for 27 months. **(B)** Multiple doses: the red circles represent the median observed data from the literature; the blue solid line presents simulated data, and the blue dotted line represents the simulated 95 percent confidence interval. A multiple-dose regimen was administered six times and observed for 72 months.

First, the KPD model for zoledronic acid was used to simulate BMD level vs. time profiles for 72 months with the same administration as the published articles. The number of simulations was 1,000. We compared the simulated results with the observed data in the article. The observed data and simulated data could be well fitted, and all the observed points were within the 95% confidence interval. This showed that our model results were robust and reliable. The results were shown in Figure 4.

**FIGURE 5:**
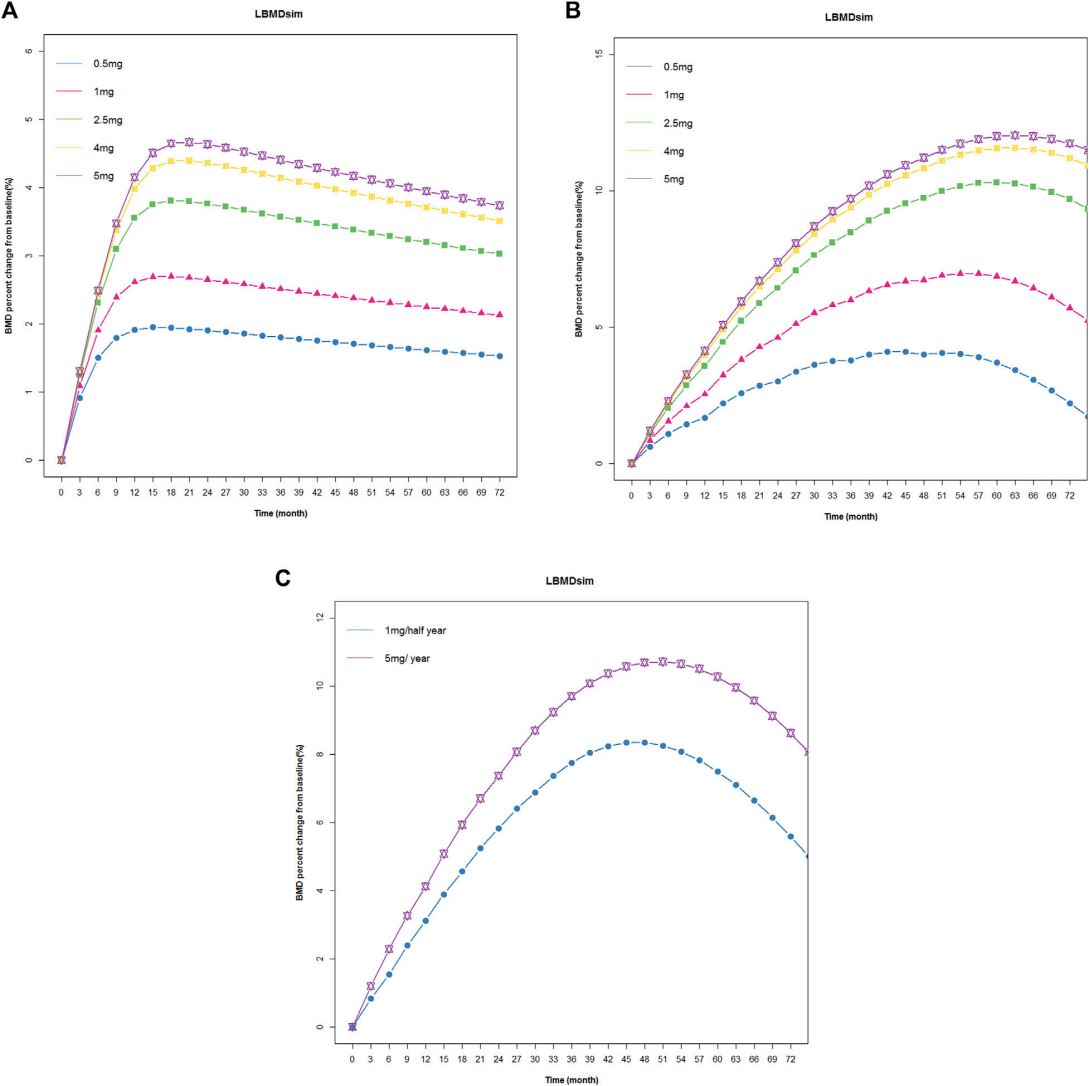
Simulated and fitted BMD profiles of zoledronic acid in patients with osteoporosis. **(A)** Single dose: patients with osteoporosis after administration a single dosing regimen, the observation lasted 72 months. **(B)** Multiple doses: patients with osteoporosis after 1 year of administration within 72 months. **(C)** Multiple doses: patients with osteoporosis after 1 mg/half year and 5 mg/year of administration, continuous treatment for 3 years.

Subsequently, we used the final model to simulate changes in BMD at 0.5 mg, 1 mg, 2.5 mg, 4 mg, and 5 mg single doses; the changes in BMD of 0.5 mg, 1 mg, 2.5 mg, 4 mg, and 5 mg were administered once a year within 72 months. At present, the standard treatment of zoledronic acid is 5 mg/year, continuous administration for 3 years. Therefore, we also simulated the changes in BMD after 1mg/half year and 5 mg/year, continuous treatment for 3 years. The results were shown in Figure 5. The lowest effective dose that could achieve a therapeutic effect was determined by a change in BMD of more than 3% from baseline.

The simulation results showed that BMD increased from baseline under different dosing regimens, and BMD increased in a dose-dependent manner. In a single dose, BMD of 2.5 mg, 4 mg, and 5 mg increased by more than 3% from baseline. In the case of once-year administration, the change in BMD of 0.5 mg and 1 mg was small, and BMD decreased rapidly after the cessation of administration. Under the twice-year regimen, BMD changes of 3% were achieved. The lowest effective dose that could achieve a therapeutic effect was determined by a change in BMD of more than 3% from baseline. Based on the above criteria and the results of E-R analysis, the regimen of 1 mg administered once every 6 months for a total of six doses could not only meet the therapeutic effect in patients but also reduce the occurrence of APR and eliminate the effect of tolerance phenomenon.

## 4 Discussion

Our study utilized the KPD model and E-R analysis to recommend an effective dosing regimen that can significantly reduce the incidence of APR in the most economical form. In addition, this regimen can reduce the tolerance of the drug in the body, potentially eliminating the possibility of drug holidays.

Reduction in fracture rate is the final clinical endpoint considered when evaluating anti-osteoporotic drug therapy (Camacho et al., 2020). However, the overall low incidence of fractures (approximately 0.3%) would make clinical trials large, expensive, and risky (Cummings and Melton, 2002). Moreover, the occurrence of fractures is affected by many factors, including the bone itself and external factors such as falls. Currently, BMD is considered as the gold standard for monitoring and diagnosing osteoporosis and has been used to predict fracture risk in many clinical applications (Fonseca et al., 2014). Bone strength includes bone mass and BMD. BMD is closely related to bone strength and is the best predictor of fracture risk. The BMD test can accurately detect the specific content of bone minerals in the body and can diagnose the symptoms of bone mineral deficiency, such as osteoporosis, to effectively carry out nutritional intervention and treatment. Bone is mainly divided into cortical bone and cancellous bone in structure. The surface area of cancellous bone is larger than that of cortical bone (Vajda et al., 2001). The primary mineralization of cancellous bone is more common, and the amount of cancellous bone in the lumbar spine is greater. The bone turnover rate of the lumbar spine is stronger than that of the hip, femur, etc. The bone mineral density of the lumbar spine after the treatment of osteoporosis drugs is better than that of other bones (Wakolbinger et al., 2019). Therefore, lumbar spine mineral density is often used as a surrogate index to evaluate the efficacy of anti-osteoporosis drugs.

The KPD model is a simplification of the classical pharmacokinetic/pharmacodynamic (PK/PD) model. The KPD model relies on dose and PD observations only (Jacqmin et al., 2007). The features of the zoledronic acid PK-PD system, with the large difference in the time course of zoledronic acid PK (hours) and the PD (months) response, suggested that the KPD model might be appropriate to describe the PD profiles of zoledronic acid (Grey et al., 2014; Shiraki et al., 2017). Moreover, the KPD model also reduces the running time.

Zoledronic acid has a residual effect, that is, the drug is deposited on the surface of the bone and can still be released into the blood after the drug is discontinued, continuing to exert an anti-bone resorption effect. A 6-year extension study randomized patients who had received zoledronic acid for 3 years to continue zoledronic acid for 3 years (Z6) or placebo (Z3P3) (Black et al., 2012). BMD at all sites remained unchanged in the Z6 group but decreased slightly in the Z3P3 group. The second extension study randomized patients who had received 6 years of zoledronic acid for 6 years to continue zoledronic acid for 3 years (Z9) and placebo (Z6P3) (Black et al., 2015). The results showed no significant difference in hip BMD between patients who continued to use zoledronic acid for 9 years and those who discontinued the drug after the 6th year. Therefore, our model incorporates a tolerance formula that produces dependence over time. Analysis of its possible causes suggests the following: 1) The increase in bone density is saturated and cannot increase indefinitely. 2) After long-term medication, the anti-resorption effect of zoledronic acid on osteoclasts is weakened; that is, the sensitivity of the receptors decreases, which leads to the phenomenon of tolerance. At present, the established quantitative pharmacological models of BPs do not consider the phenomenon of tolerance, but multiple doses are often used clinically. Therefore, the tolerance model is more consistent with the *in vivo* disposition of zoledronic acid.

At present, there is no exact standard for evaluating the efficacy of anti-osteoporosis drugs. The ACCP guidelines point out that after patients are treated with anti-osteoporosis drugs, BMD increases or stabilizes, and no fracture occurs during the treatment period, which can be considered as a good response to treatment (Camacho et al., 2020). After statistical analysis, the existing anti-osteoporosis drug treatment increased bone density by approximately 1%–10% within 1–2 years. The ACCP guidelines point out that the detection of bone mineral density is very important for the change in bone mass after treatment, and the change in BMD after treatment within the range of more than 3% has clinical therapeutic significance (Hodgson et al., 2003). In a study of alendronate in the treatment of postmenopausal osteoporosis, a 3% or more increase in vertebral BMD measurements was associated with a 50% relative risk reduction in fracture incidence (Bonnick, 2008). The risedronate study showed that patients with reduced BMD receiving risedronate had a significantly higher risk of vertebral fractures than patients with increased BMD (*p* = 0.003), suggesting that in reducing the risk of vertebral fractures, gaining BMD during treatment was better than losing BMD (Watts et al., 2004). Therefore, we selected a change in bone mineral density greater than 3% from baseline as an indicator of efficacy, and based on this, we selected an effective dosing regimen for zoledronic acid.

APR caused by zoledronic acid are mostly transient reactions, and the degree is mild, but the incidence rate is high. The observed incidence of APR varies from article to article. There are many clinical manifestations of APR (such as fever, myalgia, flu-like symptoms, headache, etc.), and there are many influencing factors, such as age, race, level of 25OHD3, and bisphosphonate-naïve status. Reid et al. (2010); Wark et al. (2012) showed that the incidence of APR was negatively correlated with age, and it was speculated that it may be related to the decrease in the level of γδ T cells in humans with age. Reid et al. (2010) suggested that the incidence of APR in non-Japanese Asian populations was generally higher than that in European and American populations. One possible reason for the high dose of zoledronic acid in Asian populations may be that its amount was directly related to the size of the skeleton. Bertoldo et al. (2010) showed that the incidence of APR after infusion of zoledronic acid in 25OHD3-deficient patients was significantly higher than that in nondeficient patients. This may be related to the activation of γδT cells by isopentenyl pyrophosphate, which can upregulate the expression of vitamin D receptors and decrease blood vitamin D levels. A.W. Popp et al. (Popp et al., 2017) found that APR were more likely to occur in zoledronic acid-naïve patients than in patients who had previously received zoledronic acid. This may be related to the long-term low level of γδT cells in patients with previous zoledronic acid treatment.

There are also some limitations in this study. First, after patients received the same dose of osteoporosis, there were interindividual differences in the changes in bone mineral density, which were mainly caused by differences in bone concentration after administration, while KPD could not establish the relationship between exposure and drug effect. Second, covariates (such as age, gender, weight, etc.) were not filtered into the model due to the limited amount of data, and the number of male subjects included in the model was too few. After we obtained the data from the clinical trial of zoledronic acid, we further completed the model.

## 5 Conclusion

In conclusion, the KPD model was able to describe the changes in BMD over time at different doses and establish a quantitative relationship between dose and BMD. The E-R analysis showed that the incidence of APR was dose dependent. The model and the E-R analysis provided a reference for dose selection.

## Data Availability

The original contributions presented in the study are included in the article/Supplementary Material, further inquiries can be directed to the corresponding authors.
