# Supplementary material for: Optimization of the dosage regimen of zoledronic acid with a kinetic-pharmacodynamic model and exposure-response analysis
Source: Front Pharmacol. 2023 Sep 27;14:1089774. doi: 10.3389/fphar.2023.1089774 (PMC10565503; doi:10.3389/fphar.2023.1089774)
Supplement: Supplementary file 1 [file Table1.DOCX]

Supplementary Material

#
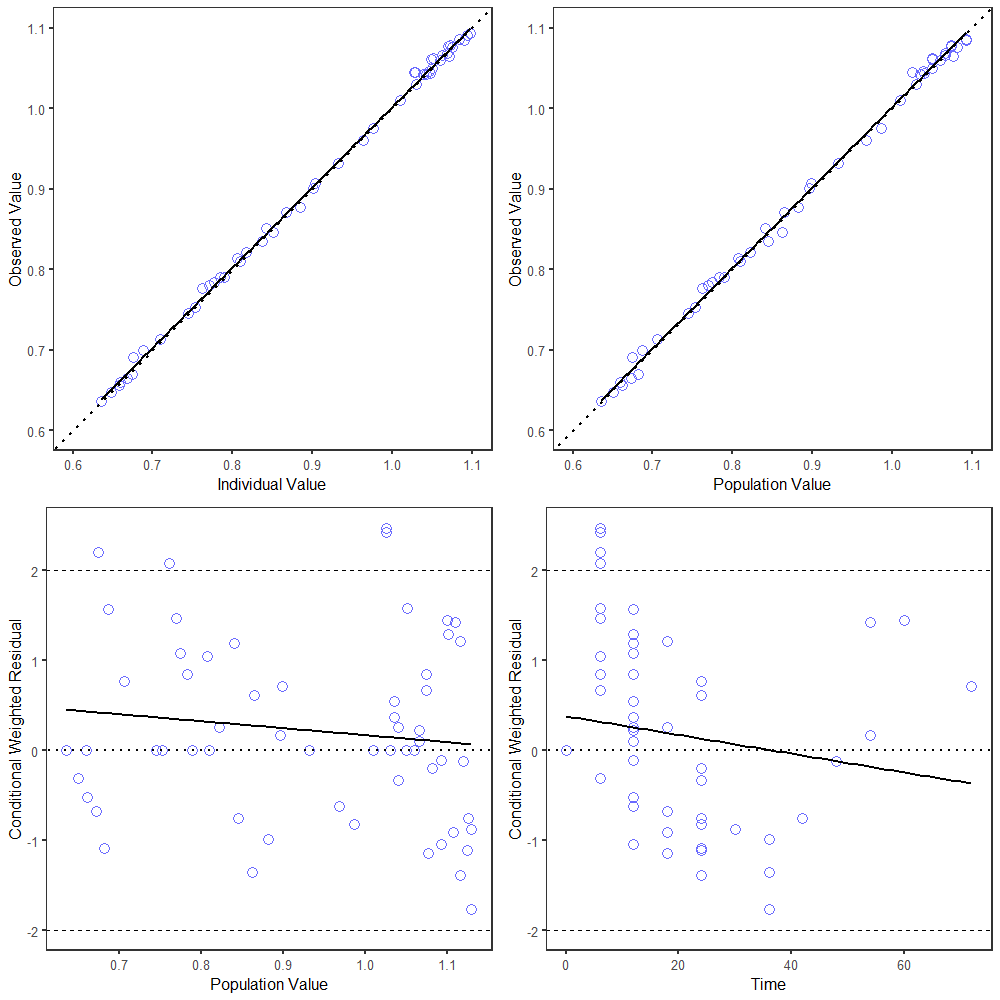
Supplementary Figures


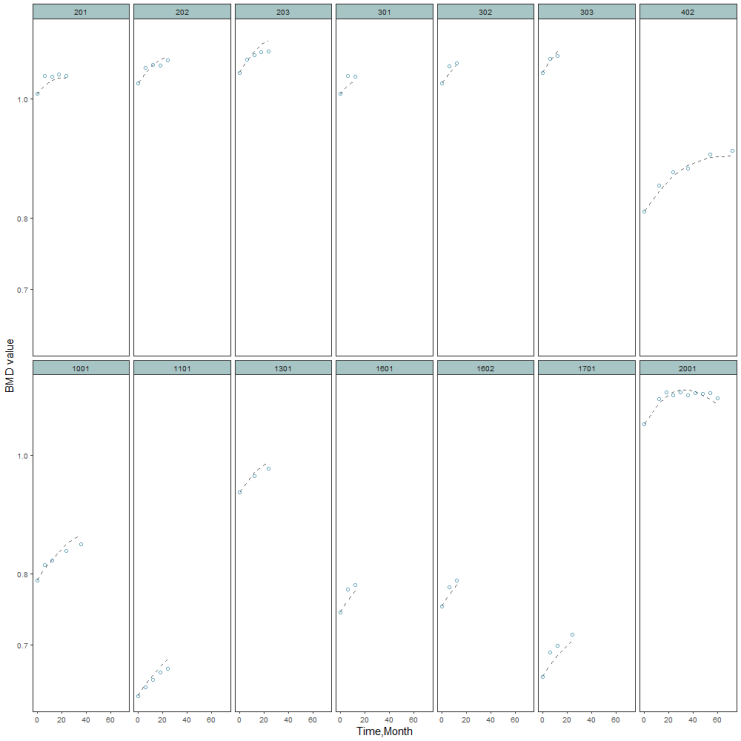
**Supplementary Figure 1.** Goodness of fit plots for the final KPD model. The blue hollow circles represent the observed data from the literature. The red solid line represents a linear smooth line. The dashed diagonal lines (top panels) and horizontal lines (bottom panels) are the lines of identity, zero lines, and conditional standardized residual (equal ± 2), respectively.

**Supplementary Figure 2.** PRED plots for the final KPD model. The blue hollow circles represent the observed data from the literature. The black line represents population prediction data.


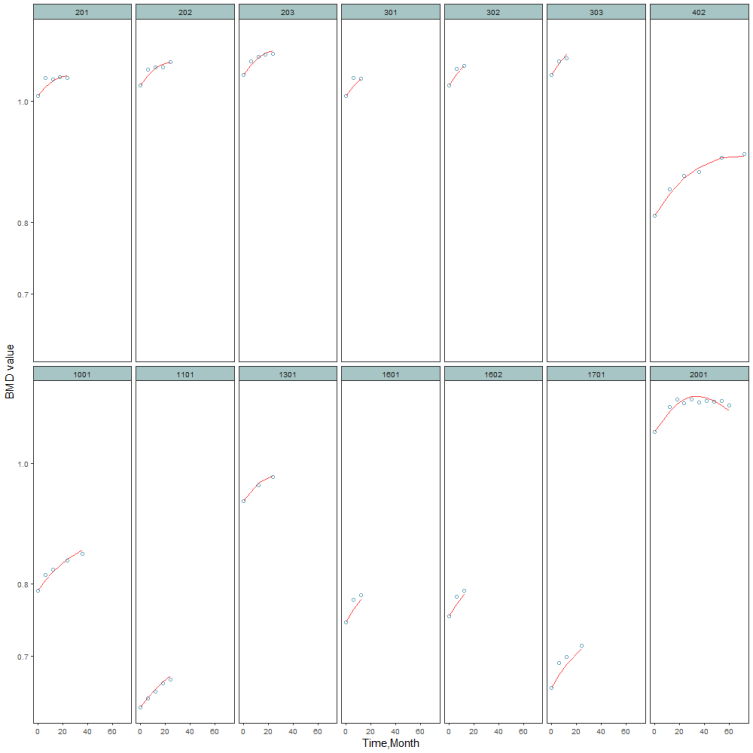


**Supplementary Figure 3.** IPRED plots for the final KPD model. The blue hollow circles represent the observed data from the literature. The red line represents individual prediction data.

**
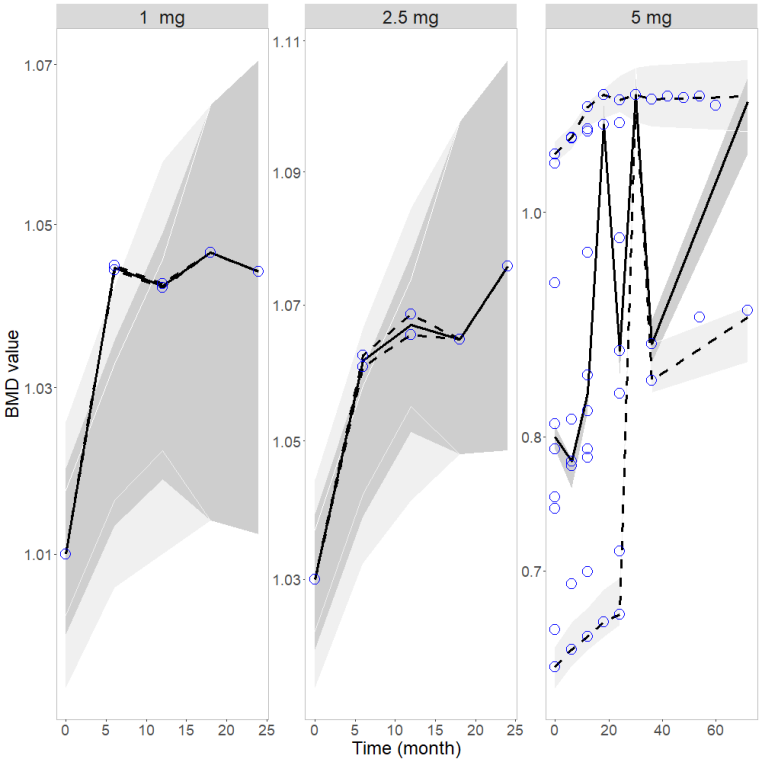
**

**Supplementary Figure 4.** Visual predictive checks (VPCs) of the final model. The blue circles represent drug concentration data. The upper black dotted line, the middle black solid line, and the lower black dotted line represent the 95th, 50th, and 5th percentiles of the observed data. The shade represents confidence intervals of the median and 5th and 95th percentiles of predictions, respectively.
